# Green and Efficient Lithium Extraction from Spent NCM Batteries via Electromagnetic Radiation

**DOI:** 10.3390/ma18173975

**Published:** 2025-08-25

**Authors:** Ling Tong, Gui-Rong Zhang, Da-Shuai Li, Xing-Yu Huang, Yuan-Long Liu, Yan-Qing Cheng

**Affiliations:** 1 School of Automation Engineering, University of Electronic Science and Technology of China, Chengdu 611731, China; zhagngr@gmail.com (G.-R.Z.); hxy000420@163.com (X.-Y.H.); 2 Yangtze Delta Region Institute (Huzhou), University of Electronic Science and Technology of China, Huzhou 313001, China; dashuai_li@csj.uestc.edu.cn; 3 Zhejiang Tianneng Advanced Material Co., Ltd., Huzhou 313100, China; longlaw@sina.com; 4 Tianneng Battery Group Co., Ltd., Huzhou 313000, China; chengyq@tianneng.com

**Keywords:** spent NCM-LIBs, black mass, electronic–magnetic radiation

## Abstract

The conventional recycling of spent lithium-ion batteries (LIBs) is hindered by high energy consumption and severe environmental pollution. In this study, a novel method utilizing high-frequency electromagnetic radiation was proposed to process the black mass derived from spent NCM-LIBs, significantly reducing both energy consumption and chemical reagent usage. Conductive carbon black was introduced as an electromagnetic-wave-absorbing additive to improve the electromagnetic energy into thermal energy conversion efficiency during electromagnetic radiation. As a result, the decomposition and reduction of NCM materials can be completed within just 10 min at a microwave power of 500 W. Following electromagnetic irradiation, lithium was efficiently extracted via simple water leaching, achieving an extraction efficiency of 88.24%. Furthermore, a microwave heating device based on traveling-wave propagation was developed. Unlike conventional small-scale microwave systems that employ resonant cavities, this design enables improved heating uniformity, higher efficiency, and greater scalability for industrial microwave-assisted chemical processes.

## 1. Introduction

Over the past decade, the application of lithium-ion batteries (LIBs) has grown exponentially, particularly in electric vehicles and energy storage systems. By 2025, global demand for LIBs is projected to surpass several hundred gigawatt-hours (GWh), accounting for approximately 70% of the rechargeable battery market [1,2,3]. Despite their widespread deployment, LIBs typically have a service life of only 5 to 8 years, resulting in a rapid accumulation of spent batteries in recent years. This trend poses a significant environmental burden and underscores the urgent need for efficient and sustainable recycling strategies. The cathode materials in NCM (LiNi_x_Co_y_Mn_z_O_2_) type LIBs contain valuable metals such as lithium (Li), nickel (Ni), cobalt (Co), and manganese (Mn). With the rapidly growing demand for LIBs, the global supply of these critical metals is facing increasing pressure. Forecasts suggest that the demand for nickel, cobalt, manganese, and particularly lithium may soon outpace known global reserves [4,5]. To mitigate this challenge, it is imperative to develop efficient strategies for recovering these metals from spent LIBs, thereby supporting a sustainable and secure supply chain for future battery production.

With the rapid expansion of LIB production and application, research on recycling technologies for spent LIBs has grown substantially in recent years. Numerous academic and industrial efforts have been devoted to this field, leading to significant advances in both fundamental studies and practical implementations. Several laboratory-scale innovations and industrial-scale technologies have already been successfully developed, reflecting promising progress in overcoming the challenges associated with LIB recycling. Current recycling methods primarily include pyrometallurgy, hydrometallurgy, and direct regeneration techniques [2,6,7,8]. Pyrometallurgy, a widely used industrial method, recovers metal alloys such as Ni, Co, and Mn through high-temperature smelting (typically above 1400 °C) followed by refining [3,9]. However, its high energy consumption raises both ecological and economic concerns [10,11]. Hydrometallurgy involves dissolving cathode materials in acidic or alkaline leaching solutions at relatively low temperatures, enabling the recovery of metals in the form of salt precursors that can be reused in new cathode synthesis [3,12,13,14]. While this method is simpler and more energy-efficient than pyrometallurgy, it requires large quantities of strong acids or alkalis, posing significant environmental and safety risks [3]. Moreover, the strong chemical bonds between high-valence transition metals (Ni, Co, Mn) and oxygen in the cathode hinder efficient leaching [1,15]. Direct regeneration restores the electrochemical performance of spent cathode materials by replenishing lithium, offering a simpler and more environmentally friendly alternative to conventional recycling methods. However, this approach remains largely limited to the laboratory scale due to challenges in scaling up for industrial production.

Given the limitations of current recycling methods—including high energy consumption, environmental risks, and poor scalability—there is an urgent need to develop sustainable technologies for processing spent NCM-LIBs. An ideal recycling strategy should achieve low energy consumption, minimal environmental impact, and high industrial feasibility. To meet these criteria, this study proposes a novel technology based on high-frequency electromagnetic wave radiation for the treatment of spent NCM-LIBs. This method seeks to address the limitations of traditional pyrometallurgical and hydrometallurgical methods by enabling the efficient and environmentally friendly recovery of valuable metals. In contrast to conventional pyrometallurgy, it leverages the interaction between electromagnetic waves and materials to achieve rapid and selective heating. When high-frequency electromagnetic waves irradiate a material, the absorbed energy is converted into thermal energy through dielectric and/or conductive losses. Unlike conventional furnace-based heating, this energy transfer occurs throughout the material’s volume due to the penetration depth of the electromagnetic waves, enabling uniform volumetric heating. The temperature increase in the material is primarily attributed to two mechanisms: (1) dipolar polarization, where high-frequency electromagnetic fields induce oscillation of polar molecules, generating heat through molecular friction; and (2) ionic or conductive loss, in which the movement of free ions or electrons under the field leads to Joule heating. Compared to conventional roasting, electromagnetic wave heating enables a much more rapid temperature increase, as energy is deposited directly and uniformly throughout the material’s volume. Unfortunately, NCM materials exhibit low dielectric loss and therefore absorb only a small fraction of electromagnetic wave energy. In contrast, amorphous carbon serves as an efficient microwave absorber due to its high electrical conductivity and disordered structure. In this study, various weight percentages of acetylene black (AB) were mixed with the black mass (BM) from spent NCM-LIBs, serving as an electromagnetic-wave-absorbing material. Upon exposure to electromagnetic radiation, the temperature of AB rapidly increased, and the generated thermal energy was transferred to the NCM material in BM. When the local temperature reached a critical point, the ionic bonds and lattice structure within the NCM materials began to break down. Previous studies on microwave carbothermal reduction without the addition of microwave-absorbing materials employed a resonant cavity system with a total input power of 1000 W and a processing time of 10 min [1]. However, such a configuration is often associated with higher energy consumption and more complex system design. In contrast, the present work used a lower input power (500 W) and a more readily engineered traveling-wave cavity, resulting in a 115 % increase in lithium recovery via simple water leaching.

In this study, we investigated the decomposition behavior of NCM materials under electromagnetic wave irradiation by varying both the atmospheric conditions and the amount of AB. The optimal atmosphere and AB dosage were identified. Furthermore, water leaching experiments demonstrated that the BM samples treated using this method achieved a high lithium extraction rate, significantly outperforming traditional furnace-based heating approaches [16]. Overall, this work presents a rapid, environmentally friendly, and energy-efficient alternative for the recycling of spent LIBs.

## 2. Experimental Section

### 2.1. Raw and Regents

The BM of spent NCM-LIBs (abbreviated as NCM-BM) used in this study was obtained by disassembling and sieving commercial spent NCM-LIBs collected from electric vehicles, provided by Zhejiang Tianneng Advanced Material Co., Ltd., Huzhou, China. The composition of the samples is summarized in Table 1. All other reagents used in the experiments were of analytical grade and commercially available.

### 2.2. Method

In this work, AB was used as an electromagnetic-wave-absorbing additive to enhance the electromagnetic energy absorption of the original NCM-BM. Upon exposure to high-frequency electromagnetic-wave radiation, AB efficiently converted electromagnetic energy into thermal energy, rapidly increasing the overall temperature of the NCM-BM to above 500 °C. At elevated temperatures, the NCM materials decomposed to form Li_2_O and other metal oxides. These metal oxides were subsequently reduced by AB, releasing CO_2_, which then reacted with Li_2_O to form lithium carbonate (Li_2_CO_3_), as shown in Equation (1) [17,18].

(1) $$\begin{array}{c} 4LiNi_{x}Co_{y}Mn_{z}O_{2}+3C+\left(2x\alpha +2y\beta +2z\gamma \right)O_{2}\overset{\mathrm{EM}\;\mathrm{radiating}}{\to }2Li_{2}CO_{3}+CO_{2}\\ +4xNiO_{\alpha }+4yCoO_{\beta }+4zMnO_{\gamma } \end{array}$$

where α, β, and γ can be 0.

After electromagnetic radiation treatment, the soluble lithium species in the samples were extracted using deionized water. The lithium leaching efficiency was calculated according to Equation (2).

(2) $$\eta_{Li}=\frac{c_{Li}V_{1}}{m_{0}w_{Li}}$$

where $c_{Li}$ represents the concentration of lithium in the leaching solution (in g/L), $V_{1}$ represents the volume of the leaching solution (in mL), $m_{0}$ represents the mass of the sample (in mg), and $w_{Li}$ represents the mass percentage of lithium in the sample (in wt.%).

### 2.3. Experimental Procedure

A total of 2 g of NCM-BM was thoroughly mixed with varying amounts of acetylene black (AB). The resulting powder mixture was loaded into a quartz boat, which was then placed along the longitudinal axis of the waveguide and subjected to electromagnetic irradiation for 10 min. The output power of the microwave generator was set to 500 W, while the power flux density received by the sample section was approximately 12.56 W/cm^2^. Following electromagnetic irradiation, the samples were leached with deionized water to extract lithium carbonate, using a solid-to-liquid ratio of 10 g/L. The slurry was stirred for 1 h at room temperature using a magnetic stirrer, then separated by high-speed centrifugation. The supernatant containing dissolved lithium carbonate was collected, while the remaining solid precipitate consisted of nickel, cobalt, and manganese oxides, graphite, residual NCM, and others.

### 2.4. Experiment Device

**Figure 1:**
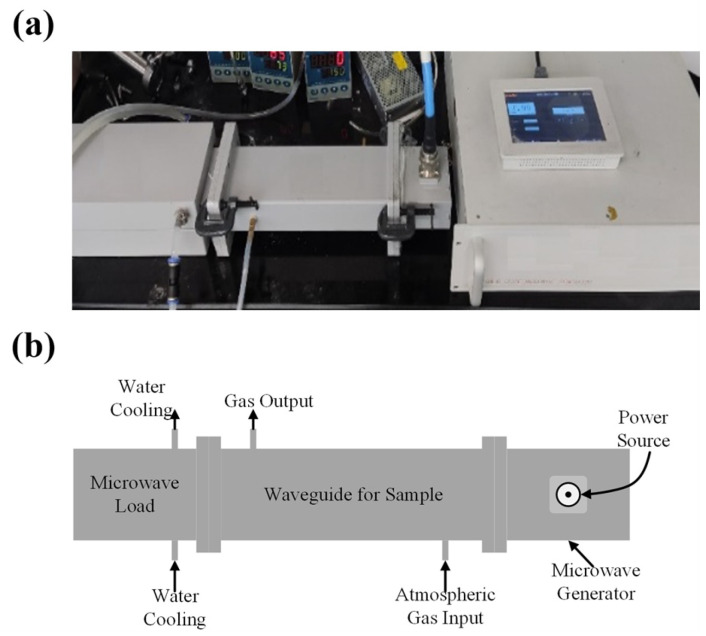
Photograph (**a**) and schematic diagram (**b**) of the EM wave system used for processing NCM-BM.

The experimental setup consisted of a high-power electromagnetic system operating at 2.45 GHz, comprising the following: (1) a hollow waveguide (dimensions: 86.36 mm × 43.18 mm × 150 mm), through which electromagnetic waves propagate along the longitudinal axis; (2) a high-power electromagnetic wave generator; (3) a coupler connecting the waveguide to the generator; and (4) a load for impedance matching, as illustrated in Figure 1. The BM samples, placed in quartz boats, were aligned along the waveguide axis, where they were uniformly irradiated by the traveling electromagnetic waves.

This waveguide-based design offers two main advantages: high energy efficiency for sample irradiation and straightforward scalability for large-scale processing. The system utilized electromagnetic radiation at 2.45 GHz, a frequency that has been demonstrated to be effective in this application.

### 2.5. Characterization

X-ray diffraction (XRD, MiniFlex 600, Rigaku, Tokyo, Japan) was employed to characterize the crystal structures of the original BM and the samples after electromagnetic irradiation. XRD in the 10° < 2*θ* < 90° with 10° min^−1^, and Cu-Kα radiation (40 kV, 15 mA) was used as X-ray source. Scanning electron microscopy coupled with energy-dispersive X-ray spectroscopy (SEM-EDS, Phenom Pharos G2, Thermo Fisher, Waltham, MA, USA) was used to examine the particle morphology and elemental distribution of the original BM and the samples after electromagnetic irradiation and water leaching. The concentrations of metal elements in both the samples and the leachate were determined using inductively coupled plasma optical emission spectrometry (ICP-OES, 5800, Agilent, Santa Clara, CA, USA) to evaluate the lithium leaching efficiency.

## 3. Results and Discussion

### 3.1. The Crucial Role of Reducing Atmosphere

**Figure 2:**
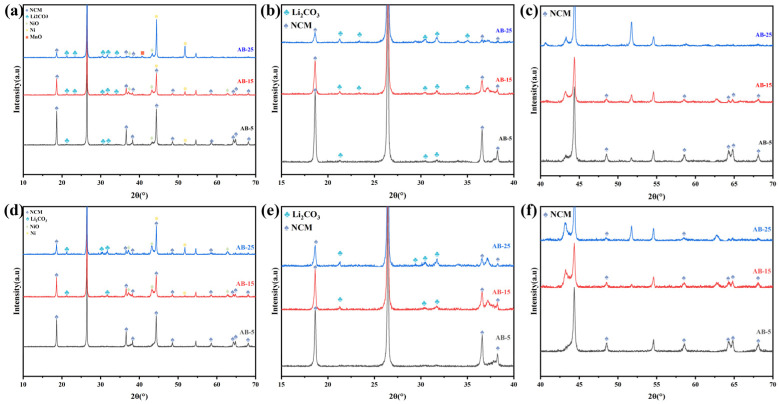
XRD patterns of the products after EM irradiation: (**a**–**c**) closed air atmosphere; (**d**–**f**) flowing air atmosphere.

**Figure 3:**
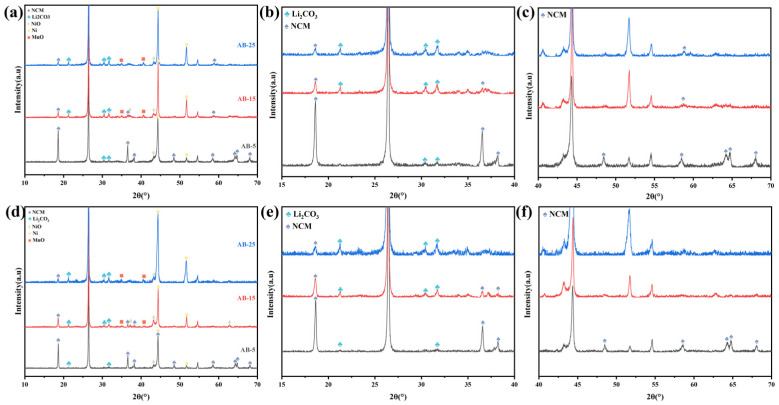
XRD patterns of the products after EM irradiation: (**a**–**c**) closed argon atmosphere; (**d**–**f**) flowing argon atmosphere.

To determine the optimal reaction atmosphere and appropriate amount of acetylene black (AB), comparative experiments were conducted using varying AB contents under both air and argon environments. Specifically, 5 wt.% (denoted as AB-5), 15 wt.% (AB-15), and 25 wt.% (AB-25) of AB were mixed with the original NCM-BM and subjected to electromagnetic wave irradiation under four conditions: closed air atmosphere, flowing air atmosphere (300 sccm), closed argon atmosphere, and flowing argon atmosphere (300 sccm). The XRD patterns of the resulting samples are presented in Figure 2 and Figure 3.

As shown in Figure 2a–d and Figure 3a–d, the intensity of the Li_2_CO_3_ peaks (2*θ* = 31.78°) consistently increases with the amount of AB added, regardless of the atmospheric condition, while the intensity of the NCM peaks (2*θ* = 18.73°) correspondingly decreases. These results indicate that AB addition facilitates the decomposition of NCM and the subsequent formation of Li_2_CO_3_, which is favorable for enhancing lithium extraction during water leaching. This promoting effect can be attributed to the excellent electromagnetic wave absorption capability of AB. Under electromagnetic irradiation, AB absorbs energy via conductive loss and efficiently converts it into thermal energy. As a result, the AB particles, uniformly distributed within the BM, act as localized heat sources, enabling rapid and uniform heating of the surrounding NCM particles. Furthermore, a higher AB content leads to greater energy absorption and elevated sample temperatures, thereby promoting more complete decomposition of the NCM.

Furthermore, as shown in Figure 2, samples treated in a closed air environment (Figure 2a,b) exhibit slightly stronger Li_2_CO_3_ diffraction peaks and weaker NCM peaks compared to those treated in a flowing air atmosphere. This suggests that the decomposition and reduction of NCM proceed more effectively in the closed system. The difference can be attributed to the oxygen availability: in the flowing air environment, the continuous supply of oxygen suppresses the formation of a reducing atmosphere, whereas in the closed air system, limited oxygen is gradually consumed. Once depleted, a locally reducing environment forms within the waveguide. This interpretation is further supported by the significantly higher diffraction intensity of the NiO characteristic peaks (2*θ* = 43.29°) observed in the flowing air condition, indicating insufficient reduction under excess oxygen.

As shown in Figure 3, the NCM diffraction peaks of the samples treated in a closed argon atmosphere are slightly weaker than those in a flowing argon environment, whereas the Li_2_CO_3_ peaks are correspondingly stronger. This subtle difference can be attributed to the cooling effect of the flowing argon gas, which removes a portion of the generated heat during electromagnetic irradiation. Consequently, the sample temperature in the flowing argon condition is slightly lower, leading to a reduced extent of NCM decomposition and lithium carbonate formation.

**Figure 4:**
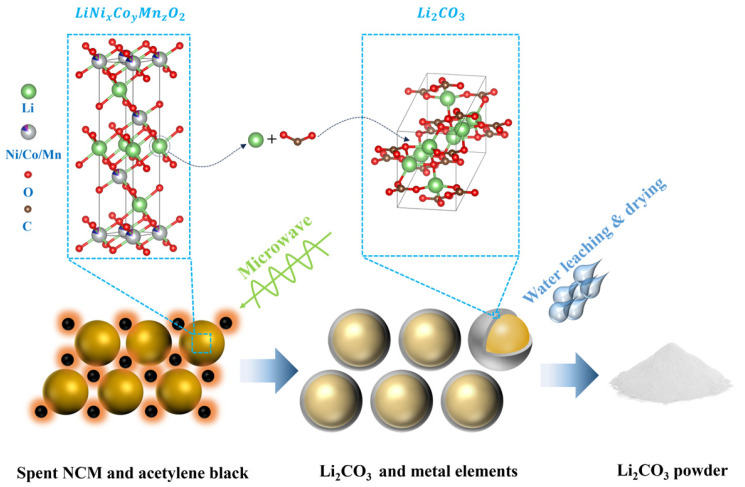
Schematic illustration of Li_2_CO_3_ formation in BM under electromagnetic irradiation and its extraction via water leaching.

**Figure 5:**
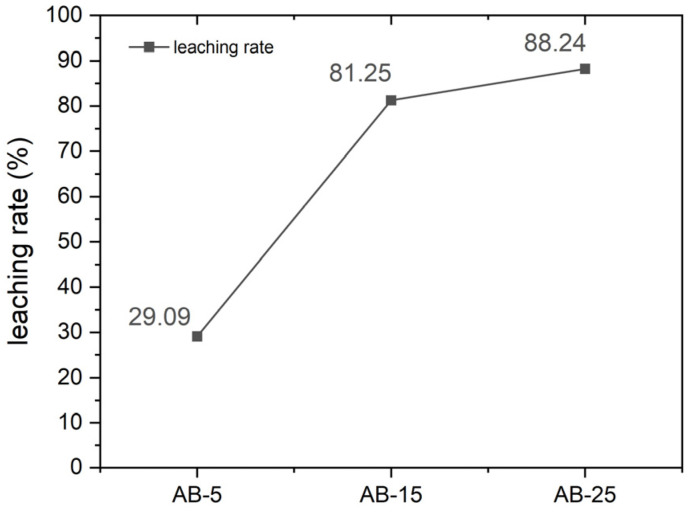
Relationship between lithium leaching rate and the amount of AB added during the water leaching process.

Therefore, based on the above results, it can be concluded that the addition of AB significantly promotes the decomposition of NCM and the formation of Li_2_CO_3_ during electromagnetic irradiation, with the process being more effective under a reducing atmosphere. As illustrated in Figure 4, during electromagnetic wave irradiation, AB within the sample absorbs electromagnetic energy and efficiently converts it into thermal energy, enabling uniform heating of the surrounding NCM particles. A higher AB content leads to increased absorption of electromagnetic energy and greater heat generation, thereby facilitating more complete decomposition of NCM and enhanced formation of Li_2_CO_3_. This effect is further confirmed by the subsequent water leaching process. As shown in Figure 5, the lithium extraction rate increases progressively with increasing AB content, reaching a maximum of 88.24%. The leaching samples were obtained from experiments conducted in a closed argon atmosphere.

### 3.2. Formation of Lithium Carbonate and Its Recovery via Water Leaching

**Figure 6:**
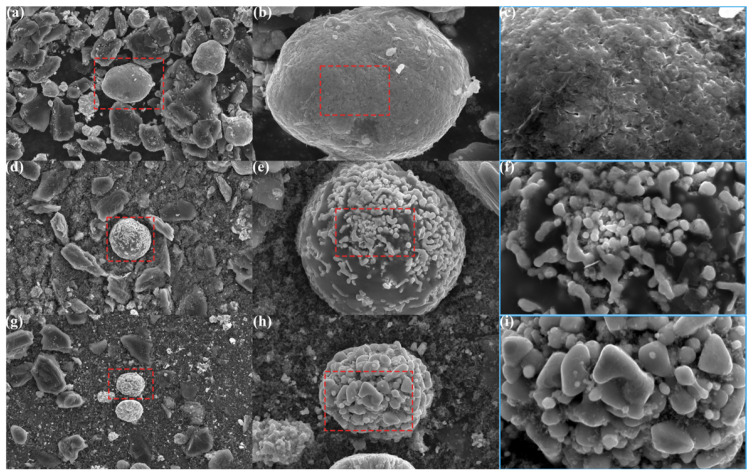
Morphologies of particles at different stages of the process. (**a**–**c**) NCM particles in the original BM; (**d**–**f**) sintered metal oxide particles mixed with Li_2_CO_3_ in the irradiated product; (**g**–**i**) sintered metal oxide particles in the residue after water leaching.

**Figure 7:**
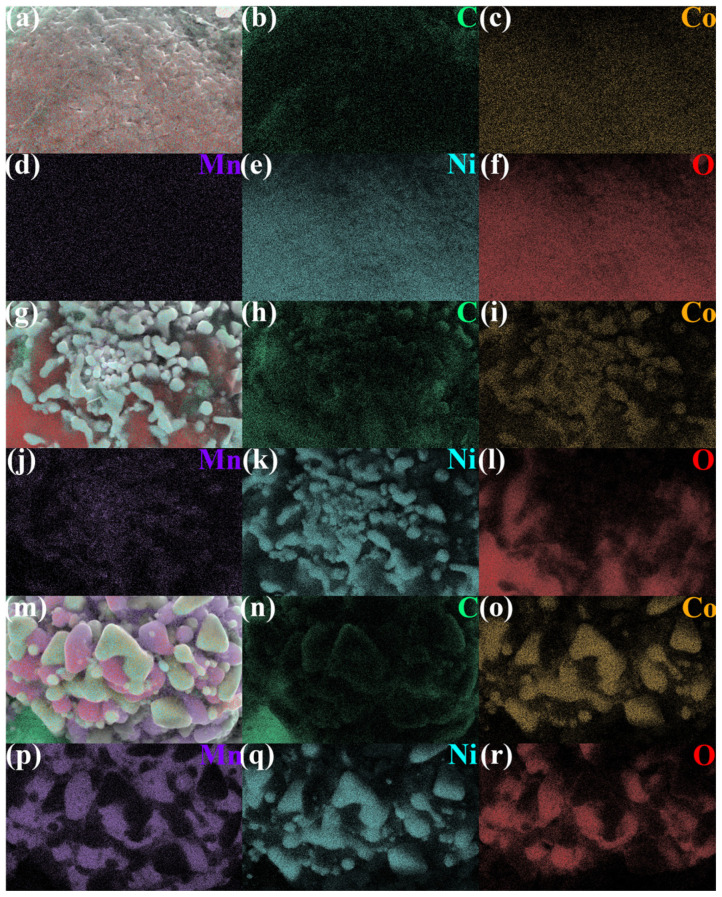
Elemental distributions on particle surfaces. (**a**–**f**) NCM particles in the original BM; (**g**–**l**) sintered metal oxide particles mixed with Li_2_CO_3_ in the irradiated product; (**m**–**r**) sintered metal oxide particles in the residue after water leaching.

To verify the high efficiency of lithium extraction via water leaching in this method, SEM-EDS analysis was performed to investigate the morphological and elemental changes of NCM particles in the original BM, the irradiated product, and the residue after water leaching. As shown in Figure 6a–c, the NCM particles in the original BM consist of numerous densely packed primary particles. The corresponding elemental mapping (Figure 7a–f) reveals a uniform distribution of nickel, cobalt, and manganese oxides on the particle surfaces. Following electromagnetic wave irradiation, the crystal structure of NCM collapses, resulting in the sintering of nickel, cobalt, and manganese metals and their oxides. Lithium oxide reacts with CO_2_ to form Li_2_CO_3_, which covers the surfaces of the sintered metal oxide particles, as observed in Figure 6d–f. The elemental distribution (Figure 7g–l) confirms this, showing bright blocky particles corresponding to metal sintering regions, and dark, ketchup-like areas adhering to them identified as water-soluble lithium carbonate. After water leaching, as shown in Figure 6g–i, the Li_2_CO_3_ is removed, leaving behind bare metal particles. The corresponding elemental mapping (Figure 7m–r) indicates that these particles are composed of sintered metals and their oxides. Therefore, the morphological evolution of NCM particles clearly reveals that the formation of Li_2_CO_3_ on the surfaces of metal particles during electromagnetic wave irradiation is the key factor enabling the high-efficiency extraction of lithium via water leaching. This finding further highlights the advantages of the proposed method.

Table 2 shows the elemental composition of the leachate obtained from water leaching of samples produced by electromagnetic wave irradiation under a closed argon atmosphere. The lithium concentration in the solution is remarkably high, whereas the concentrations of other metals, such as nickel, cobalt, and manganese, remain below the detection limit (0.0001 g/L). This result indicates the exceptional purity of Li_2_CO_3_ in the leachate, which can be attributed to the fact that electromagnetic wave irradiation generates soluble Li_2_CO_3_ as the primary product, while the transition-metal oxides remain insoluble (Figure 2 and Figure 3). Consequently, water leaching demonstrates outstanding selectivity for lithium extraction.

Furthermore, Table 3 presents the elemental composition of the residue obtained from water leaching of samples treated by electromagnetic wave irradiation under a closed argon atmosphere. The lithium content in the residue is negligible, whereas nickel, cobalt, and manganese remain at relatively high levels. These results further demonstrate that the proposed method enables efficient and selective lithium extraction.

### 3.3. Evaluation of CO_2_ Emissions and Energy Consumption

Furthermore, to highlight the potential advantages of the proposed method for industrial applications, a hypothetical analysis of CO_2_ emissions and energy consumption during the electromagnetic wave irradiation process was carried out.

During electromagnetic wave irradiation, the major mass loss of the sample arises from oxygen released by the collapse of the NCM lattice and from the oxidation of carbon (primarily acetylene black, AB). Therefore, the mass change of the sample before and after irradiation under a closed argon atmosphere was recorded (Table 4) to provide a straightforward estimation of CO_2_ emissions generated during the irradiation process.

As shown in Table 4, with increasing amounts of AB, the mass loss first rises rapidly and then increases more gradually. This trend can be explained by the fact that a higher AB content facilitates the conversion of electromagnetic energy into thermal energy during irradiation, thereby elevating the sample temperature and accelerating the decomposition and reduction of NCM. Under these conditions, the mass loss mainly originates from the decomposition of NCM and the consumption of carbon. Consequently, the mass loss increases sharply at lower AB contents. However, the amount of NCM in the sample is finite, and further addition of AB only strengthens the reducing atmosphere within the waveguide and promotes the reduction of transition-metal oxides, without causing additional decomposition of NCM. As a result, the mass loss no longer increases rapidly at higher AB additions.

Based on this analysis, it can be assumed that when 25 wt.% AB is added, the mass loss after electromagnetic irradiation is entirely attributable to the consumption of carbon. Based on this assumption, for each ton of Li_2_CO_3_ recovered, approximately 0.55 tons of CO_2_ are released during the irradiation process. This value is substantially lower than that of conventional production of Li_2_CO_3_ from lithium spodumene, which generates about 20.4 tons of CO_2_ per ton of Li_2_CO_3_ produced [19].

Furthermore, during electromagnetic wave irradiation, the cross-sectional area of the sample is approximately 2.5 cm^2^, and the energy flux density near the cross-section is about 12.56 W/cm^2^. Accordingly, the electromagnetic power absorbed by the sample is estimated to be ~31.4 W. Based on this value, the energy consumption is calculated to be 11,315 MJ (3143 kWh) for recovering one ton of Li_2_CO_3_, and 9420 MJ (2617 kWh) for processing one ton of spent LIBs BM. These values are not only far lower than those of conventional production of Li_2_CO_3_ from spodumene (218,000 MJ per ton of Li_2_CO_3_), but also significantly below the energy demand of previous furnace-based recovery methods (Table 5).

Taken together, these results highlight that the proposed electromagnetic irradiation process achieves both markedly reduced CO_2_ emissions and substantially lower energy consumption compared with conventional methods, demonstrating its exceptional potential for sustainable large-scale applications.

## 4. Conclusions

This work presents a novel recycling method for the black mass (BM) of spent NCM-based lithium-ion batteries (NCM-LIBs) using electromagnetic radiation. Upon irradiation, the NCM in the BM undergoes decomposition, resulting in the formation of soluble lithium salts (Li_2_CO_3_). During the subsequent water leaching process, a lithium extraction rate of up to 88.24% was achieved. This method is rapid, energy-efficient, and environmentally friendly, eliminating the need for chemical reagents such as acids or bases. Furthermore, the treatment system utilizes a waveguide design in which electromagnetic waves propagate in a traveling-wave mode, ensuring a more uniform field distribution. Compared with traditional resonant cavity systems, this approach offers greater scalability and practical advantages for industrial applications. In summary, this study provides a promising and sustainable strategy for LIB recycling and supports the advancement of circular economy practices in energy storage technologies.

## Figures and Tables

**Table 1:** Elemental composition of NCM-BM (wt.%).

Ni	Co	Mn	Li	Al	Cu
24.38	4.19	3.42	3.42	0.46	0.41

**Table 2:** Elemental concentrations in the leachate (g/L).

Sample	Li	Ni	Co	Mn	Al	Cu
AB-5	0.2330	ND [a]	ND	ND	0.0062	ND
AB-15	0.6383	ND	ND	ND	0.0055	ND
AB-25	0.6984	ND	ND	ND	0.0086	ND

[a] Not detected.

**Table 3:** Elemental concentrations in the residue (wt.%).

Sample	Li	Ni	Co	Mn	Al	Cu
AB-5	2.89	25.30	4.62	3.58	0.48	0.35
AB-15	0.83	26.85	4.86	3.77	0.56	0.45
AB-25	0.50	25.92	4.69	3.63	0.48	0.50

**Table 4:** Mass loss of NCM–AB mixtures after electromagnetic irradiation under a closed argon atmosphere.

Sample	Original Mass (mg)	Mass Loss (mg)	Mass Loss Rate
AB-5	2094.46	45.96	2.2%
AB-15	2304.19	186.83	8.1%
AB-25	2498.15	254.81	10.2%

**Table 5:** Time and energy consumption of traditional methods.

Reference	Original BM	Method	Time (min)	Energy [a] (MWh/t)
[20]	NCM	furnace-based	30	~1000
[21]	NCM	furnace-based	180	~6000
[22]	NCM	furnace-based	30	~1000
Our work	NCM and Graphite	EM irradiation	10	~2.6

[a] The weights of the samples were set at 1.0 g. The powers of the thermal furnaces were set at 2000 W.

## Data Availability

The original contributions presented in this study are included in the article. Further inquiries can be directed to the corresponding author.
